# Matrix-Assisted Laser Desorption Ionization-Time of Flight Mass Spectrometry Identification of Yeasts Is Contingent on Robust Reference Spectra

**DOI:** 10.1371/journal.pone.0025712

**Published:** 2011-10-13

**Authors:** Angie Pinto, Catriona Halliday, Melissa Zahra, Sebastian van Hal, Tom Olma, Krystyna Maszewska, Jonathan R. Iredell, Wieland Meyer, Sharon C.-A. Chen

**Affiliations:** 1 Centre for Infectious Diseases and Microbiology Laboratory Services, Westmead Hospital, Sydney, New South Wales, Australia; 2 Department of Microbiology and Infectious Diseases, Sydney South West Pathology Service, Liverpool, Sydney, New South Wales, Australia; 3 Molecular Mycology Research Laboratory, Westmead Millenium Institute, Sydney Medical School – Westmead Hospital, University of Sydney, Sydney, New South Wales, Australia; Charité, Campus Benjamin Franklin, Germany

## Abstract

**Background:**

Matrix-assisted laser desorption ionization-time of flight mass spectrometry (MALDI-TOF MS) for yeast identification is limited by the requirement for protein extraction and for robust reference spectra across yeast species in databases. We evaluated its ability to identify a range of yeasts in comparison with phenotypic methods.

**Methods:**

MALDI-TOF MS was performed on 30 reference and 167 clinical isolates followed by prospective examination of 67 clinical strains in parallel with biochemical testing (total n = 264). Discordant/unreliable identifications were resolved by sequencing of the internal transcribed spacer region of the rRNA gene cluster.

**Principal Findings:**

Twenty (67%; 16 species), and 24 (80%) of 30 reference strains were identified to species, (spectral score ≥2.0) and genus (score ≥1.70)-level, respectively. Of clinical isolates, 140/167 (84%) strains were correctly identified with scores of ≥2.0 and 160/167 (96%) with scores of ≥1.70; amongst *Candida* spp. (n = 148), correct species assignment at scores of ≥2.0, and ≥1.70 was obtained for 86% and 96% isolates, respectively (vs. 76.4% by biochemical methods). Prospectively, species-level identification was achieved for 79% of isolates, whilst 91% and 94% of strains yielded scores of ≥1.90 and ≥1.70, respectively (100% isolates identified by biochemical methods). All test scores of 1.70–1.90 provided correct species assignment despite being identified to “genus-level”. MALDI-TOF MS identified uncommon *Candida* spp., differentiated *Candida parapsilosis* from *C. orthopsilosis* and *C. metapsilosis* and distinguished between *C. glabrata*, *C. nivariensis* and *C. bracarensis.* Yeasts with scores of <1.70 were rare species such as *C. nivariensis* (3/10 strains) and *C. bracarensis* (n = 1) but included 4/12 *Cryptococcus neoformans*. There were no misidentifications. Four novel species-specific spectra were obtained. Protein extraction was essential for reliable results.

**Conclusions:**

MALDI-TOF MS enabled rapid, reliable identification of clinically-important yeasts. The addition of spectra to databases and reduction in identification scores required for species-level identification may improve its utility.

## Introduction

Yeast infections cause significant mortality in critically ill and immunocompromised patients. In particular, *Candida* species are the 4^th^ most common cause of nosocomial bloodstream infections in the United States, and *Cryptococcus neoformans*, the commonest cause of fungal meningitis worldwide [1], [2]. Whilst *Candida albicans* remains the leading pathogenic yeast, infections due to non-*C. albicans* species such as *Candida glabrata*, as well as previously rare opportunists such as *Trichosporon* and *Geotrichum* species, are increasingly reported [1], [3]–[6]. Novel pathogenic *Candida* species such as *Candida nivariensis* and *Candida bracarensis* have also been described [7]. Since many non-*C. albicans Candida* and non-*Candida* yeasts are resistant or less susceptible to antifungal agents, rapid accurate species identification is central to timely, effective antifungal therapy [3]–[6], [8], [9].

Conventional phenotypic-based methods for yeast identification, however, are slow (24–72 h), insensitive and often unable to identify more unusual species. Various molecular techniques including real-time PCR, DNA sequence analysis, microarray analysis, and fluorescence *in-situ* hybridization provide accurate identification [10], [11] but are expensive, require substantial specimen processing time (hours to a day) and are not easily implemented as routine techniques in the clinical laboratory.

Matrix-assisted laser desorption ionization-time of flight mass spectrometry (MALDI-TOF MS) has emerged as a powerful and rapid tool for the identification of bacterial and yeast pathogens [12]–[17]. Using MALDI-TOF MS, the protein spectral “fingerprint” of an isolate is compared to a reference spectral database for identification [18]. Previous studies have reported species identification rates of 92–99% amongst collections of yeasts and yeast-like organisms [14]–[17], [19], [20]. However, taken collectively, the results may not be directly comparable since these studies have used different approaches to assign species – from comparing spectra from test organisms to reference spectra in MALDI-TOF MS databases [16], [20], to enhancing these databases, and in some instances developing study-specific databases, with “in-house” spectral signatures [14], [15]. There also is a continuing need to extend the repository of reference spectra in MALDI-TOF MS databases.

We therefore undertook the present study to evaluate the utility of the MALDI Biotyper 2.0 Microflex LT spectrometer (Bruker Daltonik GmbH; Bremen, Germany) with its current spectral database in comparison with phenotypic-based methods for the identification of a broad range of yeasts in a diagnostic laboratory. The first part of the study comprised testing of reference and clinical strains from our culture collection. The second was a blinded prospective analysis of freshly-collected yeast isolates recovered during routine laboratory work flow; discrepant results between MALDI TOF MS and phenotypic methods were resolved using sequence analysis of the fungal internal transcribed spacer (ITS) regions [21], [22]. Results obtained by preparation of yeasts by extraction of fungal proteins and by direct application of yeast colonies onto the MALDI TOF MS plate were also compared.

## Methods

### Yeast isolates

A total of 264 yeast isolates were studied. These included 30 reference strains (26 species) obtained from the American Type Culture Collection (ATCC; Rockville, MD) and the Centraalbureau voor Schimmelcultures (CBS; Utrecht, The Netherlands) (Table 1) and, 167 clinical isolates (26 species) from the culture collections at the Centre for Infectious Diseases and Microbiology Laboratory Services, Westmead Hospital; and the Department of Microbiology, Liverpool Hospital, Sydney, Australia (Table 2).

**Table 1:** MALDI TOF MS identification of 30 reference yeast strains.

Isolates	MALDI-TOF Identification according to Bruker Score*		
	<1.70	≥1.7-<2.0	≥2.0
***Candida spp.***			
*Candida albicans* CBS 562			*C. albicans*
*Candida albicans* ATCC 10231			*C. albicans*
*Candida catenulata* CBS 565			*C. catenulata*
*Candida ciferii* CBS 4856#	No ID (*Candida orthopsilosis*)		
*Candida colliculosa* CBS 133		*C. colliculosa*	
*Candida dubliniensis* CBS 7987			*C. dubliniensis*
*Candida glabrata* ATCC 90030			*C. glabrata*
*Candida guilliermondii* CBS 2030			*C. guilliermondii*
*Candida haemulonii* CBS 5149			*C. haemulonii*
*Candida kefyr* ATCC 4135			*C. kefyr*
*Candida krusei* ATCC 6258			*C. krusei*
*Candida lipolytica* CBS 6124			*C. lipolytica*
*Candida lusitanae* CBS 5030			*C. lusitanae*
*Candida metapsilosis* ATCC 96144			*C. metapsilosis*
*Candida norvegensis* CBS 1922			*C. norvegensis*
*C. orthopsilosis* ATCC 96139		*C. orthopsilosis*	
*Candida parapsilosis* CBS 604			*C. parapsilosis*
*Candida parapsilosis* ATCC 22019			*C. parapsilosis*
*Candida rugosa* CBS 613		*C. rugosa*	
*Candida tropicalis* CBS 94			*C. tropicalis*
*Candida utilis* CBS 1600			*C. utilis*
*Candida utilis* ATCC 9950			*C. utilis*
*Candida zeylanloides* CBS 619	No ID (*C. zeylanoides*)		
***Other yeasts***			
*Arxiozyma telluris§* CBS 2685	No ID (*Candida sloofii§*)		
*Arxiozyma telluris* CBS 2676	No ID (*C. krusei*)		
*Cryptococcus neoformans* ATCC 00112			*C. neoformans*
*Cryptococcus gattii* ¶ ATCC 32608			*Filobasidiella bacillisporus* ¶
*Debaryomyces carsonii* CBS 2285#	No ID (*C. neoformans*)		
*Pichia kluyveri* CBS 3082#	No ID(*C. krusei*)		
*Pichia ohmeri* CBS 5367		*P. ohmeri*	

ID, identification; species designated within brackets refers to closest match species.

*Results from testing of four replicates.

# Reference species-specific spectra not contained in software (version 3.1.2.0, Bruker Daltonik GmbH).

¶ *A. telluris* synonymous with *C. sloofii*; *C. gattii* synonymous with *F. bacillisporus*.

**Table 2:** Species and genus identification of clinical yeast isolates by MALDI-TOF MS.

Yeast species(final ID)*	No. tested	No ID(score <1.7)	No. with genus level ID (score ≥1.7)	% genus ID	No. with species level ID (score >2.0)	% species ID
**Retrospective study**						
***Candida spp.***	***148***	***6***	***142***	***96***	***127***	***86***
*Candida albicans*	25	0	25	100	25	100
*Candida bracarensis*	1	1	0	0	0	0
*Candida dubliniensis*	6	0	6	100	3	50
*Candida famata*	2	0	2	100	2	100
*Candida glabrata*	27	0	27	100	25	93
*Candida guilliermondii*	11	1	10	91	9	82
*Candida haemulonii*	1	0	1	100	1	100
*Candida krusei*	10	0	10	100	10	100
*Candida lusitaniae*	12	0	12	100	10	83
*Candida nivariensis*	10	3	7	70	5	50
*Candida orthopsilosis*	1	0	1	100	1	100
*Candida parapsilosis*	17	1	16	94	15	88
*Candida sphaerica*	1	0	1	100	1	100
*Candida tropicalis*	14	0	14	100	14	100
*Candida utilis*	10	0	10	100	6	60
***Non-Candida yeasts***	***19***	***1***	***18***	***95***	***13***	***68***
*Arxiozyma telluris*	1	1	0	0	0	0
*Cryptococcus gattii*	1	0	1	100	0	0
*Cryptococcus neoformans*	5	0	5	100	2	40
*Geotrichum candidum*	1	0	1	100	1	100
*Geotrichum silvicola*	1	0	1	100	1	100
*Lipomyces orientalis*	9	0	9	100	8	89
*Lodderomyces elongisporus*	1	0	1	100	1	100
**Prospective study**						
*C. albicans*	18	0	18	100	17	94
*Candida catenulata*	1	0	1	100	1	100
*C. dubliniensis*	2	0	2	100	2	100
*C. glabrata*	26	0	26	100	22	85
*C. kefyr*	1	0	1	100	0	0
*C. parapsilosis*	7	0	7	100	6	86
*C. tropicalis*	1	0	1	100	1	100
*C. krusei*	1	0	1	100	1	100
*C. neoformans*	6	4	2	33	1	17
*C. gattii*	1	0	1	100	1	100
*Geotrichum capitatum*	1	0	1	100	1	100
*Rhodotorula mucilaginosa*	1	0	1	100	0	0
*Saccharomyces cerevisiae*	1	0	1	100	0	0

*All discrepant identifications between MALDI-TOF MS and biochemical methods were resolved by ITS sequencing [21], [22].

For the second part of the study, 67 consecutive (57 *Candida*, and 10 non-*Candida*, yeasts) isolates (Table 2) isolated from a variety of clinical specimens over a 2-month period were prospectively identified by MALDI-TOF MS in parallel with conventional biochemical methods. All isolates were subcultured onto Sabouraud's dextrose agar (SDA; Difco Laboratories; Detroit; MI) for 48 hours at 30°C to ensure purity prior to testing.

### Standard biochemical identification

All clinical isolates in the first part of the study were identified by the API ID 32C (bioMerieux, Hazelwood, MO) or RapID Yeast Plus systems (Remel Products, Lenexa, KS), with recommended additional tests where appropriate [23], [24]. Phenotypic identification of isolates in the prospective study was performed using one or more of the following tests: (i) *Candida* isolates - germ tube test, appearance on CHROMagar™ *Candida* (CHROMagar™. Paris, France), RapID Yeast Plus or API ID 32 or Vitek 2 (bioMerieux) identification systems; (ii) cryptococcal isolates – urease assimilation, API ID 32 system, and appearance on canavanine glycine bromothymol blue agar [25]. All other yeasts were identified by the RapID Yeast Plus and/or API ID 32 identification systems.

### DNA extraction, PCR amplification and DNA sequencing

Sequencing of the ITS 1, 5.8S rRNA and ITS 2, or the D1–D2, regions of the 28S subunit of the rRNA gene, was performed as required to verify species identity of reference strains (Table 1) whilst ITS sequencing was undertaken in all cases of discrepant identifications or “no identifications” obtained by MALDI-TOF MS and phenotypic identification methods [21], [26]. DNA extraction was performed using the High Pure DNA Template Preparation kit (Roche Diagnostics, Mannheim, Germany), according to the manufacturer's instructions.

PCR amplification of the entire ITS (ITS1, 5.8S, and ITS2) region was performed as previously described using the primers ITS1 and ITS4 [22]. PCR products were purified using the GFX PCR DNA and Gel Band Purification kit (GE Healthcare UK Limited; Little Chalfont, Buckinghamshire) and commercially sequenced using the ITS 1 primer and the BigDye Terminator v. 3.1 cycle sequencing kit in the ABI PRISM 3100 genetic analyzer (Applied Biosystems, Foster city, CA). Sequences were edited using Chromas v. 2.23 software (Technelysium Pty. Ltd.) and entered into a BLASTN search [27] provided by GenBank (http://blast.ncbi.nlm.nih.gov/Blast.cgi) for species identification. A percent similarity (identity) score of ≥98% between the unknown (query) sequence and the database sequence of closest match was used as the criterion to classify an isolate to species level [28].

### MALDI-TOF mass spectrometry

After incubation of test strains for 48 h at 30°C on SDA, 2–3 yeast colonies were suspended in 300 µL molecular grade deionized water and vortexed. Next, 900 µL of 100% ethanol (Sigma-Aldrich, St Louis, MO) was then added, vortexed, and centrifuged at 15 000 g for 2 min; the supernatant was decanted and the centrifugation repeated once. The pellet was dried at 25°C and reconstituted in equal volumes of 70% formic acid (Sigma-Aldrich) and acetonitrile (Sigma-Aldrich) (in 50 µL volumes), mixed thoroughly, followed by centrifugation (15 000 g) for 2 min. One microliter of supernatant was spotted onto a 96-spot steel plate (Bruker Daltonik) and allowed to dry at room temperature before addition of 1 µL of MALDI matrix (a saturated solution of α-cyano-4-hydroxycinnamic acid (Bruker Daltonik)). Each sample was tested in quadruplicate to ensure reproducibility of spectra. An *Escherichia coli* protein extract test standard (Bruker Daltonik) was used to calibrate the instrument and a negative extraction control included for each run. For a subset of clinical isolates (n = 88) in the first part of the study, the direct colony method was also used whereby yeasts grown on SDA were applied directly as a thin film to the steel plate and allowed to dry at room temperature before MALDI-TOF MS analysis.

Mass spectra were generated with the Microflex LT mass spectrometer (Bruker Daltonik GmbH) using the manufacturer's protocol. Briefly, ≈240 laser shots per sample spot were acquired using the recommended instrument settings for organism identification (linear positive mode, 60 Hz laser frequency, 20 kV acceleration voltage, 16.75 kV IS2 voltage, 170 ns pulsed ion extraction delay, and 2,000 to 20,137 m/z range). Manual and automated spectrum processing and species identification were performed with the MALDI-Biotyper 3.0 application and software (version 3.1.2.0; Bruker Daltonik GmbH). The software compares acquired sample spectra to reference spectra in the provided database and compiles a list of best matching database records. Identification scores were interpreted according to the manufacturer's recommended criteria: a score of ≥2.0 indicated species-level identification, a score of 1.70–1.999 indicated identification to the genus level, and a score of <1.70, was interpreted as “no reliable identification”.

For each organism, three of four MS tests within the same category of score were required for a definitive result. Failures (ie. if < three tests were of the same score, or if “no reliable identification result” was obtained) were retested. A major error was whenever the resolved final genus identification differed from that proposed by MALDI-TOF MS while a result was a minor error when the genus identification was concordant but the species name was incorrect.

## Results

Initial experiments on 88 archived clinical isolates showed that extraction of yeast proteins (see **Methods**) was essential for accurate MALDI-TOF MS identifications. Using the direct colony preparation method, correct species-, and genus-level identifications were obtained for 14% and 69% of *C. albicans* isolates (total tested, n = 36), 35% and 74% of *C. glabrata* (n = 23 tested) and 13% and 38% of *C. lusitaniae* (n = 8 tested). Analysis of the remaining isolates inclusive of nine *C. kefyr* and 12 *C. parapsilosis* strains produced no reliable spectra. Protein extraction of yeasts was subsequently always performed.

### Reference strains

Correct species-level identification according to the manufacturer's criteria (spectral score ≥2.0) was obtained for 20 of 30 reference strains (16 species) including all common *Candida* species - *C. albicans*, *C. glabrata*, *Candida tropicalis*, *Candida parapsilosis* and *Candida krusei* [1], [3]) -, *C. neoformans* and *Cryptococcus gattii* (Table 1). One isolate each of *Candida colliculosa*, *Candida orthopsilosis*, *Candida rugosa* and *Pichia ohmeri* was resolved to “genus-level” ie. score of >1.70 (but <2.0) yet yielded the correct final species designation. MALDI-TOF MS distinguished between *C. parapsilosis*, *Candida metapsilosis* and *C. orthopsilosis* although identification of the last was to “genus-level” (Table 1).

There were no minor errors and no misidentifications (major errors). However, five species (n = 6 isolates) produced spectral scores <1.70 with “no reliable identification”. Species which provided the closest spectral-based match for these isolates are shown in Table 1. Reference spectra for three species, *Candida ciferii*, *Pichia kluyveri* and *Debaromyces carsonii*, were not present in the Biotyper software. The remaining two species (*Arxiozyma telluris*, *Candida zeylanoides*) failed to match their respective species-specific reference spectra. However, for *A. telluris* CBS 2685 (Table 1), the closest spectral match identified the strain as *Candida sloofii*; both *A. telluris* and *C. sloofii* are species reduced to synonymy [29].

Overall, measured against current reference spectra in the Biotyper version 3.1.2.0 library (Bruker Daltonik GmbH), concordance with DNA sequence analysis in identification to species-, and genus-level was 67% (20/30 isolates) and 80% (24/30 isolates), respectively. For species included in the database, MALDI-TOF MS resolved 74% (20/27) of isolates to species-level identification.

### Clinical isolates: Retrospective study

#### (i) MALDI-TOF MS identifications

One hundred and forty of 167 archived isolates (84%) yielded spectra which fulfilled the criteria for species-level identification, and 160 (96%) isolates, for genus-level identification (Table 2). Collectively, *Candida* pathogens yielded a higher percentage of spectral scores of ≤2.0 (127 isolates; 86%) than non-*Candida* yeasts (68%).

The mean best (ie. closest)-match score values for *Candida* species in general, showed little intra-species variation. Table 3 summarises these data for the major *Candida* species (all with mean score values of ≥2.0). *C. nivariensis* had a lower mean score value (1.81+/−0.31; Table 3). Amongst *Candida* isolates, four *Candida utilis*, three *Candida dubliniensis*, two *C. glabrata*, two *Candida lusitaniae*, two *C. nivariensis*, and a single isolate each of *Candida gulliermondii* and *C. parapsilosis* had spectral scores of 1.7–2.0 yet the resolved species identification was correct. Thus, 142/148 or 96% of isolates were resolved to correct species at a score of ≥1.7. For non-*Candida* yeasts, MALDI-TOF MS enabled correct identification of *Cryptococcus* spp. and other yeasts including *Geotrichum* and *Lodderomyces elongisporus*. Five of 19 (26.3%) isolates including four *Cryptococcus* isolates were resolved to the correct species but with scores of 1.72–1.79 (Table 2).

**Table 3:** Variations of the match score values generated by the Biotyper system for selected *Candida* species.

Isolate	No. of isolates (tested in quadruplicate)	Mean +/− SD
*Candida albicans*	25	2.26+/−0.09
*Candida glabrata*	27	2.20+/−0.13
*Canddia krusei*	10	2.18+/−0.07
*Candida nivariensis*	10	1.81+/−0.31
*Candida parapsilosis*	17	2.19+/−0.13
*Candida tropicalis*	14	2.19+/−0.10

Abbreviations: SD, standard deviation.

#### (ii) MALDI-TOF MS versus conventional identification

For *Candida* spp., MALDI-TOF MS and phenotypic-based identification provided correct species-level identification for 127 (85.8%) and 112 (75.6%) isolates, respectively (data not shown). Of 17 discordant identifications, 16 were resolved in favour of MALDI-TOF MS using ITS sequencing. The MALDI Biotyper was able to identify *C. nivarineisis* (n = 5 isolates), *Candida famata* (n = 2), *C. lusitaniae* (n = 2) and *Candida haemulonii* (n = 1), with scores of ≥2.0, where biochemical methods yielded a result of “*Candida* spp.” Biochemical methods also erroneously classified three strains of *C. parapsilosis* as *C. lusitaniae*, and a single strain each of *C. guilliermondii*, *C. glabrata* and *Candida sphaerica* as other *Candida* species. Conversely, they identified a *C. parapsilosis* isolate that yielded no identification by MALDI-TOF.

Concordance with biochemical identification to genus level was present for 26/27 (97%) for non-*Candida* isolates (data not shown). Phenotypic methods were unable to identify *Geotrichum candidum*, *Geotrichum silvicola* and *L. elongisporus*, all successfully identified by MALDI-TOF MS, yet speciated all six *Cryptococcus* spp., of which only two yielded spectral scores of >2.0.

#### (iii) Incorrect and no identification

There were no major errors by MALDI-TOF MS. Six isolates (3.6%) yielded a result of “no identification” (score of<1.7). These were three *C. nivariensis*, one *C. bracarensis*, one *C. guilliermondii* and one *A. telluris* isolates (Table 2). Reference spectra for *C. bracarensis* are not present in the Bruker database version 3.2.1.0 (Bruker Daltonik).

### Clinical isolates: Prospective study

Table 2 summarises the identification results for 67 yeast isolates (13 species) by conventional methods, MALDI-TOF MS and the final species designation as determined by ITS sequencing. Biochemical methods identified all 67 isolates. There were no major errors in identification by MALDI-TOF MS.

Four of the six cryptococcal isolates studied were not identified by MALDI-TOF MS criteria (scores 1.3–1.5; Table 2). Assignment to species level at a score of ≥2.0 was achieved for 53 (79%) isolates (100% concordant with biochemical identification methods). Ten isolates including four *C. glabrata*, one *C. albicans* and one *C. parapsilosis*, had spectral scores of >1.70 but <2.0; single isolates of *Candida kefyr* (mean score 1.93), *Rhodotorula mucilaginosa* (mean score 1.96) and *Saccharomyces cerevisiae* (mean score 1.97) also yielded similar spectral scores (Table 2). All genus-level identifications resolved isolates to their correct species designations.

The time to issuing a definitive identification result by phenotypic tests after recovery of the isolate on culture was 24–72 h whilst the average hands-on time per isolate for MALDI-TOF MS identification after culture isolation was approximately one hour including sample preparation.

## Discussion

Whilst the utility of the MALDI-TOF MS is established for the efficient, routine identification of bacteria [12], [30], its position in assisting with, or replacing, phenotypic identification of yeasts in the clinical mycology laboratory is less well defined. The present study re-affirms and extends the results of previous reports of the ability of the Bruker Microflex LT (Bruker Daltonik) MALDI-TOF system to provide fast and accurate identification of a broad range of yeasts. Key findings were that (a) identification was genus-as well as species- dependent, (b) there were no major errors, including in the absence of reference spectra for a species, resulting in misidentifications, and (c) isolates with spectral scores between 1.70 and 1.90 were all correctly resolved.

We first validated MALDI-TOF MS for yeast identification using well-characterised reference strains in comparison with ITS sequencing. To more accurately represent the utility of the MALDI Biotyper 2.0 instrument with its current database, we did not add new spectral entries to the database. Although not all isolates achieved species-level identification using manufacturer-recommended criteria, *all* common *Candida* species [1], [3] were unambiguously assigned (Table 1). Uncommon *Candida* spp. with genus-level identifications were also resolved to their correct species and importantly, “misidentifications” only associated with scores of <1.70 and therefore not given an identification. Whilst we cannot explain why *C. zeylanoides* CBS 619 (spectra contained in the Bruker library version 3.1.2.1), was not identified, lower scores may result from poor extraction technique, inadequate drying of the yeast pellet or inadvertent inclusion of agar into the extract. However, the scores were reproducible between runs and high scores (≥2.0) were achieved for common yeasts (Tables 1, 2, 3) analysed within the same run using identical protein extraction. Lower scores for uncommon yeasts have been noted by others [14]–[16]. The ability to provide species-level identification is dependent on the number of entries per species in the manufacturer's database, usually being higher for common isolates. A higher number of entries for the same species (as for common yeasts) will better reflect diversity within the species due to possible variation in protein expression between strains [31]; such intraspecies variation in fungi which may impact on protein spectral profiles warrants further study.

Examination of a large number of clinical strains (n = 167) confirmed the observations for reference strains. When measured against ITS sequence analysis, MALDI-TOF MS performed better than biochemical tests (84% of isolates correctly identified with spectral scores of ≥2.0 vs. 76.4%). Other studies examining yeast profiles following protein extraction have noted that lowering the score threshold for species-level identification to ≥1.80 or even ≥1.70 can still be used to provide reliable identification [14], [16]. In our study, a score cut off of ≥1.70 allowed correct identification of 96% of isolates, and *Candida* isolates, lowering the threshold from ≥2.0 to ≥1.70 increased species-level identification by an additional 12%. Despite these findings, however, analyses of large numbers of different yeast species across multiple centres using standardised extraction methods are essential prior to defining a consensus cut-off score. Mean score values for common *Candida* species (eg. *C. albicans*, *C. glabrata*) were ≥2.0 with small standard deviations reflecting good result reproducibility, and likely, the number of spectra from these species already contained in the database. *C. nivariensis* yielded lower mean score values with greater variance (1.81+/−0.31); reproducibility is likely to improve with additions of reference spectra into the database (currently, n = 2 in the database). Five species of non-*Candida* yeasts were profiled with scores of ≥2.0 yet four of six *Cryptococcus* strains had scores of <2.0 (≥1.70; see below).

The Bruker database (version 3.1.2.1) used in our study was a more recent version containing a larger number of spectral entries compared with that in three other studies [14]–[16] and logically, may be expected to provide better identification rates. Possible explanations for the apparent *reduced* ability of MALDI-TOF MS to identify isolates to “species level (84%) in comparison with previous reports (87–100%) include differences in the range or mix of yeast species, and nature of the database employed. In studies where complementation of the manufacturer's database with in-house spectra or where the reference database was specifically constructed for the purpose of the study, species-level identification rates have ranged from 92.5–100% [14], [15], [32]. Others using the Bruker database without supplementation have reported similar identification rates to those observed herein [16], [20].

A novel finding was the ability of MALDI-TOF MS to distinguish within the *C. glabrata* clade ie. between *C. glabrata* and *C. nivariensis* and *C. bracarensis*, which presently rely on molecular methods for species separation [7], [33], [34]. Spectra from *C. nivariensis* were clearly distinguished from those of *C. glabrata.* Seven of 10 clinical isolates had spectral scores of >1.8 (five were >2.0; Table 2); possible reasons for the lower scores for the remaining three include suboptimal protein extraction despite every care with the manufacturer's extraction protocol, or variability in protein expression within this species. Spectra from the only isolate of *C. bracarensis* were novel. Members of the *C. parapsilosis* species complex, indistinguishable by biochemical methods [35], were also resolved by MALDI-TOF MS, including *C. parapsilosis sensu stricto*, *C. metapsilosis* and *C. orthopsilosis* [14], [15], [17]. Quiles-Melero *et al* reported that these three species as assigned by pyrosequencing, yielded MALDI-TOF MS results with 100% concordance [36]. We noted that a further member of the *C. parapsilosis* group, *L. elongisporus*, also yielded species-level identification. Differentiation of species within the *C. parapsilosis* and *C. glabrata* complexes is important since there are species-specific differences in antifungal susceptibilities and ability to form biofilms *in vitro* [34], [35], [37].

We further assessed in real time the efficiency of MALDI-TOF MS for routine yeast identification. Although only 79% of yeasts achieved species-level identification, all genus-level identification results (94%) yielded the correct final species designation. There were no major errors. Biochemical methods provided 100% species identification in this arm of the study as they identified four *C. neoformans* isolates that produced a score of <1.70 by MALDI-TOF (the “closest match” spectra still assigned the correct species). Unreliable identifications for *C. neoformans* have been reported by others (identification rates 50–66%) [14], [16] and may be due to the polysaccharide capsule of this pathogen, rendering extraction and solubilisation of proteins difficult or due to insufficient database entries to enable spectral matches. We observed for *Cryptococcus* spp., that colonies with mucoid morphology posed technical difficulties with extraction, often requiring repeat analysis. Although McTaggart *et al*. reported species-level identification rates of 100% for *Cryptococcus* spp. [32] this was only after complementation of Bruker software with home-generated spectra.

The results of the present study have also provided novel spectra for four pathogens - *C. ciferi*, *C. bracarensis*, *P. kluyveri*, and *D. carsonii -* for future integration into the Bruker library. Spectra were acquired by testing each isolate in quadruplicate to ensure good reproducibility and on two separate occasions (data not shown) but require further independent validation by testing larger numbers of isolates.

Preparation for MALDI-TOF MS analysis can be undertaken in two ways. Although the direct colony method is used to identify bacteria, our attempts to adapt this for yeasts resulted in no spectra or unacceptably low scoring spectra in a large proportion of cases including for the most common pathogen, *C. albicans*, and there is now evidence that even for bacteria, extraction methods may be necessary [38]. By adhering to the manufacturer's protocol for fungal cell wall disruption, reliable spectra were achieved in most instances. Other clues in assessing MALDI-TOF results include noting that the highest scores for species-level identification were seen when there was a large gap in score values or “score jump” between the most likely identification to the next most likely result ([39], this study).

MALDI-TOF MS offers considerable savings in time and cost over standard yeast identification methods. A single isolate may be identified in 45–60 mins including extraction time; up to 96 isolates may be identified within 3 h at AUD0.50 per sample and the protein extraction procedure is readily integrated into routine workflow. Phenotypic identification methods cost ≈ AUD10 per sample and take up to 48 h for definitive results. Costs associated with ITS sequencing where phenotypic methods cannot provide identification are ≈AUD9 per isolate (turn-around-time 48 h). The limitations of MALDI-TOF MS, however, are acknowledged. Robust reference spectra are required with regular curation. Other limitations are the high capital costs (≈AUD 250000) and inability to detect mixed cultures.

In conclusion, the rapid, accurate identification of pathogenic yeasts by MALDI-TOF MS is contingent on optimal protein extraction and upon robust reference spectra. *Candida s*pecies were reliably identified, with superior performance of MALDI-TOF MS over conventional methods. However, MALDI-TOF MS was less reliable in distinguishing between *C. neoformans* and *C. gattii* compared with biochemical methods.
